# Dynamic Transformer Based on Wavelet and Diffusion Prior Guidance for Cardiac Cine MRI Reconstruction

**DOI:** 10.3390/s26092842

**Published:** 2026-05-01

**Authors:** Bolun Zhao, Jun Lyu

**Affiliations:** School of Computer and Control Engineering, Yantai University, Yantai 264005, China; zhaobolun37@s.ytu.edu.cn

**Keywords:** cardiac cine MRI, MRI reconstruction, transformer, wavelet transform, diffusion model

## Abstract

Cardiac magnetic resonance imaging (CMR) is widely used for the diagnosis and functional assessment of cardiovascular diseases because of its noninvasive nature and excellent soft-tissue contrast. However, accelerated cine magnetic resonance imaging (cine MRI) acquisition usually relies on undersampling, which may lead to noise, aliasing artifacts, and detail loss in reconstructed images. To address this issue, we propose a wavelet-guided dynamic Transformer with diffusion priors for cardiac cine MRI reconstruction. Specifically, a diffusion model is introduced into a reduced latent feature space to generate high-frequency prior features with only 8 reverse sampling steps, thereby enhancing detail recovery while maintaining moderate computational cost. In addition, a wavelet-guided dynamic Transformer is designed to capture low-frequency structural information and temporal dependencies across adjacent frames. By combining wavelet-domain decomposition, diffusion priors, and dynamic spatiotemporal modeling, the proposed framework improves reconstruction quality while preserving temporal consistency. Experimental results on multiple cardiac cine MRI datasets show that the proposed method achieves superior reconstruction accuracy and temporal consistency over several competing approaches, while maintaining a favorable balance between computational efficiency and reconstruction performance. These findings indicate that the proposed framework is an effective and robust solution for accelerated cardiac cine MRI reconstruction.

## 1. Introduction

Cine sequences in cardiac magnetic resonance imaging (CMR) are of substantial clinical value for assessing cardiac structure and function. Short-axis cine sequences provide dynamic cross-sectional images of the heart, enabling visualization of myocardial contraction and relaxation throughout the cardiac cycle and thereby supporting quantitative evaluation of cardiac function and lesion analysis. In contrast, long-axis cine sequences depict the cardiac chambers from base to apex together with their dynamic changes, thus providing clinicians with an intuitive view of ventricular morphology and motion. However, because of the combined effects of continuous cardiac pulsation and respiratory motion, cine CMR acquisition places stringent demands on both temporal resolution and acquisition efficiency. In clinical practice, imaging is often constrained by scan time, hardware performance, and patient cooperation, which frequently leads to noise, motion artifacts, blurring, and loss of structural detail [1]. Therefore, achieving high-quality and spatiotemporally consistent cine MRI reconstruction under accelerated acquisition has become an important research focus in intelligent cardiac imaging reconstruction.

To shorten scan time, undersampling strategies are commonly adopted in clinical practice to reduce the amount of acquired k-space data. However, undersampling violates the assumptions of conventional sampling theory, leading to aliasing artifacts and structural distortions. Unlike static image reconstruction [2], which relies solely on information from a single frame, cine sequences inherently exhibit strong correlations across adjacent cardiac phases [3,4]. Although adjacent frames differ because of cardiac motion and deformation, they still preserve high anatomical consistency. Therefore, effectively exploiting the spatiotemporal complementarity across neighboring frames is essential for recovering structural details while preserving temporal coherence, and is a key step toward improving both reconstruction quality and clinical applicability in cine MRI.

In recent years, deep learning-based dynamic MRI reconstruction methods have made remarkable progress [5,6]. Many of these approaches use convolutional neural network (CNN) or recurrent architectures (RNNs) to model sequential information, thereby suppressing artifacts and improving reconstruction efficiency to some extent. However, due to their inherently local receptive fields, CNNs often struggle to capture long-range spatiotemporal dependencies in scenarios involving complex cardiac motion and fail to fully exploit nonlocal similar regions across different frames. More recently, Transformer-based methods have been introduced into dynamic reconstruction tasks [7] because of their ability to model global dependencies and enhance spatiotemporal feature representation. Nevertheless, existing Transformer-based methods still face two major challenges. First, many methods rely primarily on regression-based loss functions during training. As a result, they tend to adopt a conservative reconstruction strategy that prioritizes recovery of low-frequency structures while neglecting high-frequency details, often leading to oversmoothing in edge and texture regions. Second, when local window attention is used to reduce computational complexity, the model’s effective receptive field becomes limited [8]. This restriction may prevent the model from fully exploiting sharp and structurally similar regions in the spatiotemporal neighborhood, leading to the loss of fine-grained details and reduced coherence and accuracy in the reconstructed sequence.

Diffusion models (DMs) have recently emerged as powerful generative models for capturing high-frequency details in image and video generation tasks [9,10,11]. Through a stepwise denoising process, they can generate high-quality results rich in texture and edge information, thus providing a new opportunity for detail restoration in cine MRI reconstruction. However, directly applying DMs to cine MRI reconstruction still presents two major challenges. First, the diffusion process usually requires a large number of iterative steps, resulting in substantial computational cost and making it difficult to satisfy the efficiency requirements of clinical or near-real-time applications. Second, when input sequences are corrupted by artifacts such as undersampling artifacts or motion blur, DMs may be sensitive to these degradations and may generate details that are inconsistent with the true anatomy or even introduce structural distortions, thereby undermining the reliability of the reconstructed results.

Motivated by these considerations, this paper proposes a unified framework for cardiac cine MRI reconstruction that integrates a wavelet-guided dynamic Transformer with diffusion priors [12,13]. The proposed framework aims to achieve structurally faithful reconstruction with enhanced detail preservation, spatiotemporal consistency, and efficient inference. Wavelet transforms [14] are used to decompose the cine sequence into low-frequency structural components and high-frequency detail components, allowing the model to separately model global structures and local details. The dynamic Transformer is responsible for recovering stable anatomical structures and dominant motion patterns from the low-frequency components, while the diffusion process generates compact high-frequency prior features with fewer iterations. These priors are fused with Transformer features to restore fine textures and edge details lost under undersampling. Furthermore, a wavelet-domain spatiotemporal propagation and fusion mechanism is introduced to leverage complementary information across adjacent frames and enhance temporal coherence.

The primary contributions can be summarized as follows:

1. We propose WGDT-Diff, a unified framework for accelerating cardiac cine MRI reconstruction that integrates wavelet-domain decomposition, dynamic Transformer modeling, and diffusion-based prior guidance techniques.
2. We design a latent-space diffusion prior learning strategy capable of generating high-frequency prior features in just 8 diffusion iterations, thereby enhancing detail compensation while reducing computational cost.
3. We propose a wavelet-guided dynamic modeling and fusion design, including the Wavelet-Guided Dynamic Attention Module (WGD-AM), the Wavelet-Guided Dynamic Feed-Forward Module (WGD-FFM), and the Wavelet-Guided Bidirectional Fusion (WGBF) module, to improve inter-frame information interaction and temporal consistency in cardiac cine MRI reconstruction.
4. Experimental results on the CMRxRecon dataset and an in-house dataset demonstrate that the proposed WGDT-Diff consistently outperforms existing state-of-the-art methods in terms of reconstruction quality, detail fidelity, and temporal consistency, validating its effectiveness and robustness.

## 2. Related Works

### 2.1. Traditional MRI Reconstruction

Traditional MRI reconstruction methods mainly include parallel imaging and compressed sensing (CS)-based approaches. Representative parallel imaging methods, such as SENSE [15] and GRAPPA [16], accelerate reconstruction by exploiting receiver coil sensitivity information or self-calibrated k-space interpolation. Although widely adopted in clinical practice, their performance is often limited by coil configuration, sensitivity estimation accuracy, and noise amplification, especially at high acceleration factors. CS-based methods [17] further improved accelerated MRI reconstruction by introducing sparsity priors and nonlinear optimization. Representative methods, including k-t FOCUSS [18], L + S [19], and DLMRI [20], enhance reconstruction by modeling spatiotemporal sparsity, low-rank structure, and dictionary-based priors, respectively. However, these methods typically depend on hand-crafted priors and iterative optimization, which limits their representational capacity and computational efficiency. In cine MRI, these limitations often lead to residual artifacts, blurred details, and degraded temporal consistency.

### 2.2. Static MRI Reconstruction

Static MRI reconstruction has developed from end-to-end learning to model-driven and generative paradigms. Early methods, such as AUTOMAP [21], directly learned the mapping from undersampled measurements to reconstructed images, demonstrating the feasibility of data-driven MRI reconstruction. GAN-based methods [22,23] were later introduced to improve perceptual quality and texture fidelity, although their reconstruction faithfulness may be limited when data consistency is insufficient. To better integrate imaging physics with learned priors, deep unrolling methods became a mainstream direction. Variational Networks [24,25,26] and MoDL [27] combined iterative optimization, regularization learning, and data consistency constraints, achieving strong performance in static MRI reconstruction. More recently, Transformer-based methods such as ReconFormer [28] improved long-range dependency modeling through multi-scale attention mechanisms. Diffusion models [29] have also shown strong capability in recovering fine-grained structures and have been increasingly adopted in medical image reconstruction [30,31,32,33].

Nevertheless, these methods are mainly designed for single-image or slice-wise reconstruction. When extended to cine MRI, they often process frames independently and fail to fully exploit temporal dependencies across adjacent frames. Therefore, static MRI reconstruction methods provide an important basis, but cannot replace dynamic approaches tailored for joint spatiotemporal reconstruction.

### 2.3. Dynamic MRI Reconstruction

Dynamic cine MRI reconstruction is more challenging than static reconstruction because undersampling artifacts not only affect spatial structures but also propagate along the temporal dimension [2,34], thereby impairing motion continuity. Therefore, recent studies have shifted from frame-by-frame reconstruction to joint spatiotemporal modeling. Deep Cascade [35] combines convolutional de-aliasing and data consistency modules for dynamic cardiac MR reconstruction, demonstrating the benefit of sequence-level recovery. CRNN [5] further improves reconstruction by introducing recurrent mechanisms to model temporal information across frames and iterations. However, CNN- and RNN-based methods still have limited ability to capture long-range dependencies, which may result in insufficient detail preservation and temporal inconsistency.

More recently, Transformer-based methods such as VRT [7] have been introduced to enhance long-range dependency modeling. In parallel, diffusion-based methods have also been explored for dynamic MRI reconstruction [36]. In addition, diffusion frameworks have been extended to zero-shot dynamic MRI reconstruction [37] and to domain-conditioned temporal modeling for both Cartesian and non-Cartesian dynamic acquisitions. Nevertheless, existing diffusion-based methods still often suffer from high computational cost or rely heavily on an initial reconstruction. Therefore, jointly preserving spatial details and temporal coherence in an efficient manner remains a key challenge in dynamic cardiac MRI reconstruction.

## 3. Methodology

### 3.1. Overview

**Figure 1:**
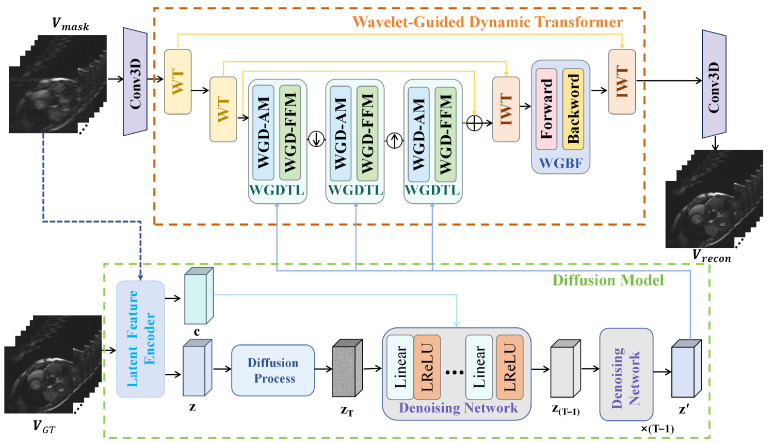
Schematic illustration of the proposed WGDT-Diff framework, which consists of the wavelet-guided dynamic Transformer (WGDT) and the diffusion model (DM). WT and IWT denote the wavelet transform and inverse wavelet transform, respectively, and ⊕ denotes element-wise summation.

Figure 1 presents an overview of the proposed WGDT-Diff framework. The framework consists of two tightly coupled components: the Wavelet-Guided Dynamic Transformer (WGDT) and a Diffusion Model (DM). The DM generates a compact latent prior feature, denoted as $z^{\prime }\in R^{T\times C^{\prime }}$, where $C^{\prime }$ denotes the channel dimension of the latent representation. Specifically, the input cine sequence is compressed into a reduced latent space whose dimensionality is much lower than that of the original image-domain input, thereby improving the efficiency of diffusion-based prior generation. The resulting compact prior feature mainly captures complementary high-frequency information and is used to guide the subsequent reconstruction process. Based on this prior information, WGDT-Diff maps the undersampled cardiac cine MRI sequence $V_{\mathrm{mask}}\in R^{T\times 2\times H\times W}$ to the reconstructed sequence $V_{\mathrm{recon}}\in R^{T\times 2\times H\times W}$.

### 3.2. Wavelet-Guided Dynamic Transformer

As illustrated in the WGDT part of Figure 1, the undersampled video frames $V_{\mathrm{mask}}\in R^{T\times 2\times H\times W}$ are first passed through a 3D convolution layer to extract shallow features, yielding the global feature map $F_{\mathrm{in}}\in R^{T\times C\times H\times W}$. Subsequently, a wavelet transform (WT) is applied to decompose these features into approximation coefficients $F_{a}\in R^{T\times C/4\times H\times W}$ and detail coefficients $F_{d}\in R^{T\times 3C/4\times H\times W}$. The approximation coefficients $F_{a}$ are then further decomposed by another WT, producing $F_{aa}\in R^{T\times \hat{C}\times H\times W}$ and $F_{ad}\in R^{T\times 3\hat{C}\times H\times W}$, where $\hat{C}=C/16$. Next, in the Wavelet-Guided Dynamic Transformer Layer (WGDTL), the reconstruction feature $F_{\mathrm{out}}$ is extracted from the approximation coefficients $F_{aa}$ and the prior feature $z^{\prime }$, while the WGBF module is employed to further explore spatiotemporal information. After feature extraction, two inverse wavelet transforms progressively fuse these features to reconstruct the artifact-free cine MRI sequence $V_{\mathrm{recon}}$. The architectures of WGDTL and WGBF are described in detail in Section 3.2.1 and Section 3.2.2, respectively.

#### 3.2.1. Wavelet-Guided Dynamic Transformer Layer

**Figure 2:**
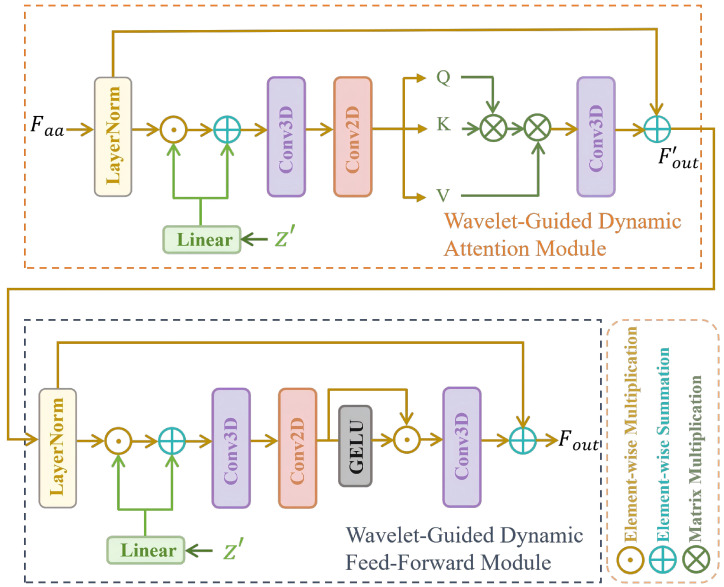
Schematic illustration of the Wavelet-Guided Dynamic Transformer Layer (WGDTL), composed of the Wavelet-Guided Dynamic Attention Module (WGD-AM) and the Wavelet-Guided Dynamic Feed-Forward Module (WGD-FFM).

As shown in Figure 2, WGDTL consists of a Wavelet-Guided Dynamic Attention Module (WGD-AM) and a Wavelet-Guided Dynamic Feed-Forward Module (WGD-FFM). In this study, multiple WGDT layers are hierarchically stacked to effectively fuse the approximation coefficients $F_{aa}$ with the prior feature $z^{\prime }$. Within the WGD-AM, given the approximation coefficients $F_{aa}$, the highly compact prior feature $z^{\prime }$ is utilized to guide the reconstruction process:

(1) $$\hat{F}=W_{l}^{1}z^{\prime }⊙LN\left(F_{aa}\right)+W_{l}^{2}z^{\prime }$$

where ⊙ denotes element-wise multiplication, LN denotes layer normalization, and $W_{l}$ represents a linear layer. Next, $\hat{F}$ is transformed into $Q=W_{2}^{Q}W_{3}^{Q}\hat{F}$, $K=W_{2}^{K}W_{3}^{K}\hat{F}$, and $V=W_{2}^{V}W_{3}^{V}\hat{F}$ using a 3D convolution and a 2D convolution, where $W_{3}$ and $W_{2}$ denote the channel-wise 3D convolution and depth-wise 2D convolution, respectively. The 3D convolution captures joint temporal-spatial information over *T*, *H*, and *W*, while the 2D convolution is identically applied to each temporal slice. The attention is then computed as follows:

(2) $$F_{out}^{\prime }=W_{a}\hat{V}\cdot Softmax\left(\hat{K}\cdot \hat{Q}/\gamma \right)+F_{aa}$$

where γ denotes a learnable scaling factor.

In the WGD-FFM module, the prior feature $z^{\prime }$ is first fused into $F_{out}^{\prime }$ to obtain ${\hat{F}}_{out}^{\prime }$. Subsequently, a 3D convolution layer is employed to aggregate temporal information, while Conv2D is used to aggregate spatial information from neighboring pixels. The overall process of WGD-FFM can be formulated as follows:

(3) $$F_{out}=G\left(W_{2}^{1}W_{3}^{1}{\hat{F}}_{out}^{\prime }\right)⊙W_{2}^{2}W_{3}^{2}{\hat{F}}_{out}^{\prime }+{\hat{F}}_{out}^{\prime }$$

where *G* denotes the Gaussian Error Linear Unit (GELU).

#### 3.2.2. Wavelet-Guided Bidirectional Fusion

Given the features ${\left\{M_{i}\right\}}_{i=1}^{N}$ produced by the first inverse wavelet transform (IWT), most existing methods exploit neighboring-frame information by stacking either the original features or their aligned counterparts. Nevertheless, direct stacking often fails to fully capture useful inter-frame correlations and may propagate inaccurate information when feature estimation or alignment is imperfect. To address this issue, we introduce a Wavelet-Guided Bidirectional Fusion (WGBF) module to enhance long-range spatiotemporal modeling while suppressing the influence of unreliable features.

**Figure 3:**
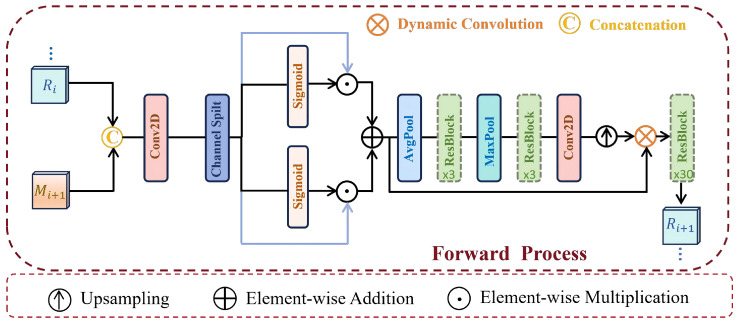
The structure of the Forward Process in the Wavelet-Guided Bidirectional Fusion.

WGBF consists of a forward branch and a backward branch. As shown in Figure 3, the forward branch is used here to illustrate the fusion process, while the backward branch follows the same architecture with the temporal propagation direction reversed. Concretely, $R_{i}$ and $M_{i+1}$ are concatenated to obtain $\bar{M}$, where $R_{i}$ is the output of the previous forward step and $M_{i+1}$ is the input of the current step. A convolution layer and a LeakyReLU activation are then applied to map $\bar{M}$ to $\hat{M}$, which is subsequently split into ${\hat{M}}_{1}$ and ${\hat{M}}_{2}$ along the channel dimension. The fused feature is finally obtained from ${\hat{M}}_{1}$ and ${\hat{M}}_{2}$ as follows:

(4) $$F_{pre}={\hat{M}}_{1}⊙S\left(W_{1}{\hat{M}}_{1}\right)+{\hat{M}}_{2}⊙S\left(W_{2}{\hat{M}}_{2}\right)$$

where *S* denotes the Sigmoid activation function, while *W* represents a 3×3 2D convolution operator. Based on this design, spatial feature extraction can be written as:

(5) $${\bar{R}}_{i+1}=F_{pre}⊗upsample\left(P\left(F_{pre}\right)\right)$$

where *P* denotes a stack composed of AvgPool, ResBlock, MaxPool, ResBlock, and Conv2D. Finally, ${\bar{R}}_{i+1}$ is passed through 30 ResBlocks to obtain $R_{i+1}$.

### 3.3. Diffusion Model

The proposed diffusion model, derived from the conditional denoising diffusion probabilistic model, comprises a forward diffusion process and a reverse denoising process. Specifically, a latent encoder (LE) [38] is adopted to extract the target latent feature $z\in R^{T\times C^{\prime }}$ from the fully sampled cardiac cine MRI sequence, and the conditional feature $c\in R^{T\times C^{\prime }}$ from the undersampled cardiac cine MRI sequence. The proposed model aims to predict the prior feature $z^{\prime }\in R^{T\times C^{\prime }}$ under the condition of $c\in R^{T\times C^{\prime }}$, where z is used as the target feature. In all experiments, the reverse diffusion process is performed with 8 reverse sampling steps (T=8), which was further validated in Section 4.6. The diffusion process is described in detail as follows:

#### 3.3.1. Diffusion Process

First, the LE extracts the prior feature z. During the forward diffusion process, this feature is gradually perturbed into a noisy version $z_{t}\in R^{T\times C}$, which can be expressed as follows:

(6) $$q\left(z_{t}|z\right)=N\left(z_{t};\sqrt{{\bar{\alpha }}_{t}}z,\left(1-{\bar{\alpha }}_{t}\right)I\right),$$

where *T* denotes the total number of diffusion steps, and N represents a standard Gaussian distribution. The parameters $\alpha_{t}$ and $\bar{\alpha }t$ are defined as $\alpha_{t}=1-\beta_{t}$ and ${\bar{\alpha }}_{t}=\prod_{i=1}^{t}\alpha_{i}$ for t=1,…,T, where the hyperparameters β1:T∈(0,1) control the noise variance at each step.

#### 3.3.2. Denoising Process

The reverse diffusion operates as a Markov chain, progressively denoising the latent features from $z_{T}$ back to $\hat{z}$. For instance, the transition from $z_{t}$ to $z_{t-1}$ can be described as follows:

(7) $$q\left(z_{t-1}|z_{t},z_{0}\right)=N\left(z_{t-1};\mu_{t}\left(z_{t},z_{0}\right),\frac{1-{\bar{\alpha }}_{t-1}}{1-{\bar{\alpha }}_{t}}\beta_{t}I\right),$$

(8) $$\mu_{t}\left(z_{t},z_{0}\right)=\frac{1}{\sqrt{\alpha_{t}}}\left(z_{t}-\frac{1-\alpha_{t}}{\sqrt{1-{\bar{\alpha }}_{t}}}\epsilon \right),$$

where ϵ denotes the noise present in $z_{t}$, which is estimated at each step by a learnable denoising network $\epsilon_{\theta }$. The previously trained LE encodes the zero-filled input cine MRI $V_{mask}$ to produce a conditional latent representation $c\in R^{T\times C^{\prime }}$. Consequently, the denoising network estimates the noise component present in $z_{t}$ based on the conditioning input c:

(9) $$z_{t-1}=\frac{1}{\sqrt{\alpha_{t}}}\left(z_{t}-\frac{1-\alpha_{t}}{\sqrt{1-{\bar{\alpha }}_{t}}}\epsilon_{\theta }\left(z_{t},c,t\right)\right)+\sqrt{1-\alpha_{t}}\epsilon_{t},$$

where $\epsilon_{t}∼N\left(0,I\right)$. After completing *T* reverse sampling steps, the diffusion model generates the predicted prior features $z^{\prime }$.

### 3.4. Training and Inference

Inspired by [39,40], the training procedure is conducted in two stages. In the first stage, the latent encoder is trained to extract the target latent features, while the wavelet-guided dynamic Transformer reconstruction network is trained for image reconstruction. In the second stage, the diffusion model is trained, based on the pretrained LE and WGDT obtained in the first stage, to generate the prior features.

#### 3.4.1. Training Phase I

In the first stage, the objective is to train the latent encoder (LE) to extract informative target prior features z from the ground-truth (GT) data. To this end, the LE and WGDT are jointly optimized as a unified framework. For a given ground-truth cine MRI sequence $V_{\mathrm{GT}}\in R^{T\times 2\times H\times W}$, the LE first encodes it into a compact set of prior features $z\in R^{T\times C^{\prime }}$:

(10) $$z=LE(V_{\mathrm{GT}})$$

These extracted features, rather than the generated feature $z^{\prime }$, are then fed into WGDT, and the diffusion model is not involved in this stage.During training, the network is optimized using the $L_{1}$ loss together with the multi-scale frequency reconstruction (MSFR) loss [41]. The formulation of the $L_{1}$ loss is given as

(11) $$L_{1}=\sum_{i=1}^{N}{∥V_{recon}^{i}-V_{GT}^{i}∥}_{1}$$

where $V_{\mathrm{recon}}^{i}$ denotes the *i*-th frame of the reconstructed cardiac cine MRI sequence, and $V_{\mathrm{GT}}^{i}$ denotes the corresponding *i*-th frame of the GT cardiac cine MRI sequence. The MSFR loss measures the L1 distance in the frequency domain between the multi-scale GT cardiac cine MRI frames and the undersampled cardiac cine MRI frames, which is defined as follows:

(12) $$L_{MSFR}=\sum_{i=1}^{N}\sum_{k=1}^{K}\frac{1}{t_{k}}{∥FT\left({\hat{S}}_{k}^{i}\right)-FT\left(S_{k}^{i}\right)∥}_{1}$$

where FT represents the Fast Fourier Transform for transforming the image signal into the frequency domain, *K* is the number of scales considered, and ${\hat{S}}_{k}^{i}$ denotes the image signal of the *i*-th frame at the *k*-th scale. Accordingly, the optimization objective of the first stage is formulated as follows:

(13) $$L_{recon}=L_{1}+\lambda L_{MSFR}$$

In this study, λ was empirically set to 0.1 to balance the image-domain reconstruction term and the auxiliary MSFR term, so as to enhance frequency-domain consistency without overwhelming the primary structural reconstruction objective.

#### 3.4.2. Training Phase II

In the second stage, for an undersampled cardiac cine MRI sequence $V_{\mathrm{mask}}\in R^{T\times 2\times H\times W}$, the trained LE is first employed to extract the conditional feature c:

(14) $$c=LE(V_{\mathrm{mask}})$$

The diffusion model (DM) is then trained to generate the prior feature $z^{\prime }$ conditioned on c. A total of *T* iterations are performed to progressively generate the prior feature $z^{\prime }$. The corresponding loss function is formulated as:

(15) $$L_{diff}={∥z^{\prime }-z∥}_{1}$$

The DM is trained to estimate $z^{\prime }$ as the prior approximation of z. Since a discrepancy between $z^{\prime }$ and z is unavoidable, the DM and WGDT are jointly optimized in this stage to better exploit complementary frequency information for cine reconstruction and to increase the robustness of WGDT to the estimated prior. The overall training loss $L_{\mathrm{total}}$ is defined as the sum of the reconstruction loss $L_{\mathrm{recon}}$ and the diffusion loss $L_{\mathrm{diff}}$:

(16) $$L_{total}=L_{recon}+L_{diff}$$

#### 3.4.3. Inference

Given an undersampled cardiac cine MRI sequence $V_{\mathrm{mask}}\in R^{T\times 2\times H\times W}$, the latent feature extractor compresses $V_{\mathrm{mask}}$ into a conditional feature $c\in R^{T\times C^{\prime }}$, while the diffusion model generates a prior feature $z^{\prime }$ from pure Gaussian noise ϵ conditioned on c. The proposed WGDT-Diff network then incorporates $z^{\prime }$ to reconstruct the undersampled cardiac cine MRI sequence.

## 4. Experiments

### 4.1. Dataset

To validate the proposed network, three single-coil cardiac cine MRI datasets were employed, including one in-house short-axis (Cine-Sax) dataset and two CMRxRecon [42] datasets with short-axis and long-axis cine sequences, respectively. The in-house training set was constructed from 27 eligible healthy volunteers, comprising 2430 images in total. Each volunteer contributed six fully sampled short-axis cine sequences, and each sequence contained 15 temporal frames. The validation and test sets were partitioned at the subject level to avoid subject overlap across subsets. During data preparation, retrospective undersampling was performed on the fully sampled k-space data using random Cartesian masks under four acceleration settings to generate the network inputs. For computational consistency, all datasets were further preprocessed before reconstruction. Specifically, the original data with a size of 512×204 were first zero-padded to 512×256 in the frequency domain and then center-cropped to 256×256 in the image domain. As the MR data are complex-valued, the real and imaginary components were concatenated as separate channels, resulting in an input dimension of [256×256×2] instead of [256×256]. For testing, short-axis (Cine-Sax*) and long-axis (Cine-Lax*) cine sequences from the CMRxRecon datasets were also adopted to evaluate the generalization capability of WGDT-Diff. Such a multi-source evaluation protocol facilitates a more comprehensive assessment of model robustness and adaptability across different clinical scenarios. The detailed data partitioning scheme is summarized in Table 1. To ensure privacy protection, all data were anonymized and de-identified prior to use.

### 4.2. Comparison Methods

We compared the proposed method with MoDL [27], KIGAN [22], SwinIR [8], DiffRecon [31], CRNN [5], and VRT [7]. These representative approaches represent different technical paradigms, including model-based deep unfolding, generative adversarial networks, Transformer-based restoration, diffusion-based reconstruction, convolutional recurrent networks, and video restoration Transformers, respectively. To comprehensively assess the reconstruction capability of the proposed method, both qualitative and quantitative evaluations were conducted.

### 4.3. Quantitative Experimental Results

Table 2, Table 3 and Table 4 present the quantitative results of WGDT-Diff and competing reconstruction methods on the in-house dataset and the CMRxRecon dataset. For both short-axis and long-axis cine sequences, WGDT-Diff achieves the best overall results across all evaluation metrics, demonstrating strong competitiveness among existing state-of-the-art methods.

Overall, while existing static reconstruction methods—such as MoDL [27], KIGAN [22], SwinIR [8], and DiffRecon [31] possess certain advantages regarding single-frame quality, they struggle to fully leverage the complementary spatiotemporal information inherent in cine sequences. Furthermore, although sequential methods such as CRNN [5] and VRT [7] incorporate temporal modeling, they still suffer from issues such as insufficient detail recovery or error accumulation under high acceleration conditions. In contrast, the proposed method achieves higher PSNR and SSIM scores, along with lower NMSE values, across all three datasets and various acceleration factors. This is accomplished through the joint modeling of sequential structural information and fine-grained details, coupled with the utilization of cross-frame information and a prior-based compensation mechanism, thereby validating its effectiveness and robustness in the context of cine MRI reconstruction tasks.

### 4.4. Qualitative Experimental Results

For qualitative analysis, the reconstructed images obtained by different methods were visually compared with the corresponding ground truth images, and error maps were further generated from the absolute differences to provide a more intuitive comparison of structural recovery, detail preservation, and artifact suppression.

**Figure 4:**
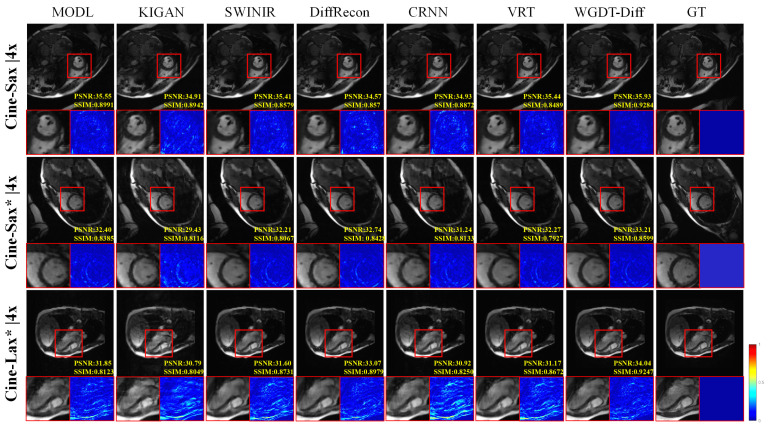
Comparative visual results on the Cine-Sax (4×) and CMRxRecon (4×) datasets are shown. The accompanying error maps are computed as the differences between the reconstructions and the corresponding cardiac-region ground truth.

**Figure 5:**
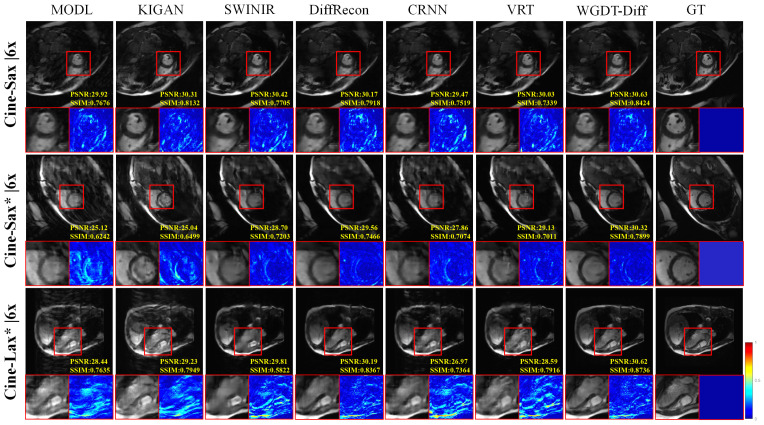
Comparative visual results on the Cine-Sax (6×) and CMRxRecon (6×) datasets are shown.

Figure 4 and Figure 5 present the qualitative results at 4× and 6× acceleration on the in-house and CMRxRecon datasets. Under these relatively low acceleration factors, most methods can recover the main anatomical structures with acceptable visual quality. Nevertheless, WGDT-Diff still shows clearer myocardial contours, more intact cardiac chamber boundaries, and fewer residual artifacts than the competing methods, indicating better preservation of fine structural details.

**Figure 6:**
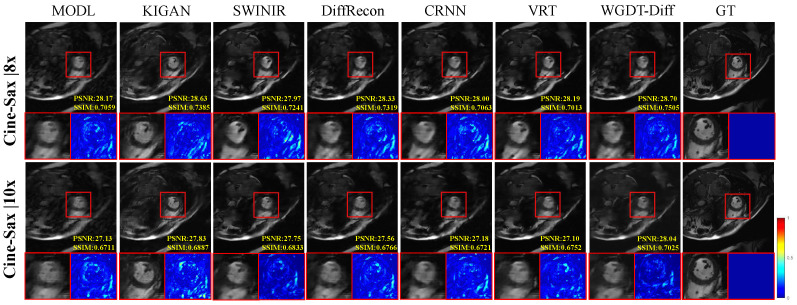
Comparative visual results on the Cine-Sax dataset at 8× and 10× acceleration are shown.

Figure 6 further shows the qualitative comparisons at 8× and 10× acceleration, where the differences among methods become more pronounced. As the undersampling ratio increases, methods such as MoDL, KIGAN, and SwinIR exhibit more obvious blurring, structural distortion, or residual artifacts, while sequential models such as CRNN and VRT still suffer from insufficient detail recovery in challenging regions. By contrast, WGDT-Diff maintains sharper anatomical boundaries, more compact local structures, and cleaner background regions. In particular, at 10× acceleration, the advantage of the proposed method becomes more evident in the highlighted regions, where WGDT-Diff produces more clearly defined structures with sharper borders and improved local contrast than MoDL, which shows more noticeable blurring and structural spreading. The corresponding error maps also show that WGDT-Diff yields more localized residuals with lower overall intensity and fewer texture residuals, indicating closer agreement with the ground truth. These observations suggest that WGDT-Diff can better preserve local structural fidelity and suppress undersampling artifacts, especially under highly accelerated acquisition.

### 4.5. Model Complexity

**Figure 7:**
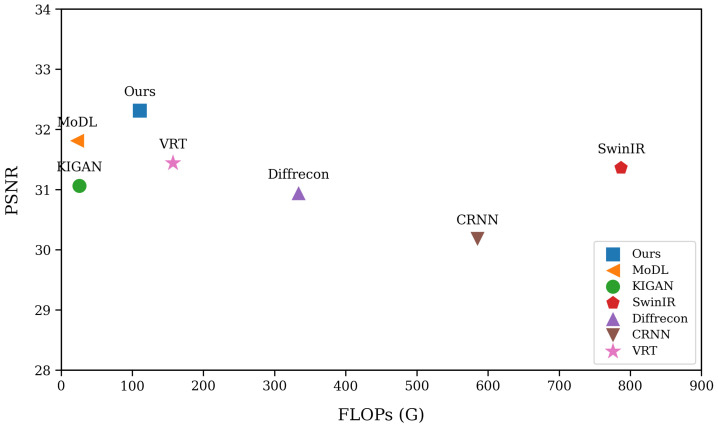
Comparison of Cine-Sax (6×) Floating-Point Operations (FLOPs) vs. Reconstruction Performance.

Table 5 further compares the methods in terms of parameter count and inference time. MoDL [27] and CRNN [5] have relatively small model sizes, showing clear advantages in lightweight design, whereas KIGAN [22], DiffRecon [31], and VRT [7] are considerably more parameter-intensive. In particular, DiffRecon [31] exhibits both the largest parameter count and the longest inference time, reflecting the heavy computational burden often associated with diffusion-based reconstruction. In comparison, the proposed WGDT-Diff contains only 12.04 M parameters and requires 305 ms per sequence for inference. Although it is slower than MoDL and VRT, which require 45 ms and 179 ms, respectively, it is still substantially more efficient than DiffRecon while delivering stronger reconstruction performance. Overall, these results indicate that WGDT-Diff offers a favorable balance between efficiency and accuracy. As shown in Figure 7, the proposed method maintains competitive reconstruction accuracy with a relatively low computational burden. It is located in the upper-left region of the graph, indicating strong reconstruction performance under relatively low FLOPs. Although some competing methods achieve competitive performance, they do so at the cost of substantially higher computational overhead, whereas others are computationally lighter but suffer from inferior reconstruction quality. This result suggests that WGDT-Diff improves reconstruction effectiveness without a disproportionate increase in computational cost, demonstrating superior efficiency overall. This advantage can be attributed to its ability to effectively exploit complementary information across adjacent frames for feature fusion, while avoiding the extra burden caused by excessive module stacking.

### 4.6. Ablation Study

**Figure 8:**
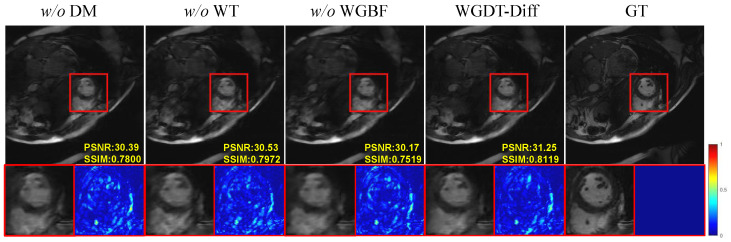
Component-wise ablation experiments on the Cine-Sax (6×) dataset.

An ablation analysis was carried out on the Cine-Sax dataset at an acceleration factor of 6× to assess the effect of each proposed component on reconstruction performance. Figure 8 presents the visual comparison results, while Table 6 summarizes the contribution of each individual module.

**Figure 9:**
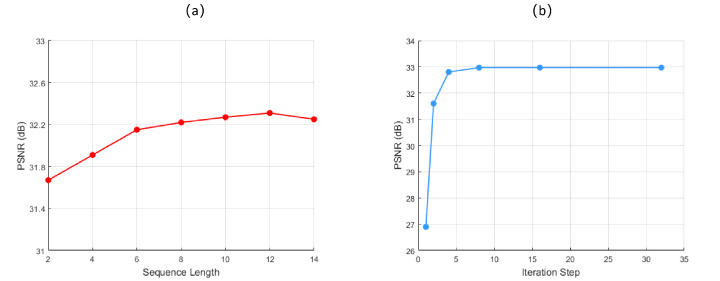
(**a**) Ablation study of the sequence length during inference. (**b**) Ablation study of the number of iteration steps.

1. The Impact of Diffusion Model. We designed a variant termed “w/o DM”, that performs reconstruction solely by relying on WGDT, without utilizing a DM to generate prior features. As shown in Table 6, the network’s reconstruction performance declined significantly in the absence of a DM, with the PSNR dropping by 1.09 dB; this demonstrates that the DM provides valuable prior features that enhance reconstruction performance.
2. The Impact of Wavelet Transforms. We conducted a comparative experiment to evaluate the effectiveness of the WT and investigate its impact on WGDT-Diff. Specifically, the WT and IWT were replaced with bilinear downsampling and upsampling, respectively. This variant is denoted as “w/o WT”, while the training strategy was kept unchanged. As shown in Table 6, the “w/o WT” variant exhibits a performance drop of 0.37 dB in terms of PSNR. These results indicate that the wavelet-based feature extraction scheme is superior to standard upsampling and downsampling methods.
3. The Impact of WGBF. To investigate the effectiveness of WGBF, this study designed a variant model termed the “w/o WGBF” model by removing WGBF from WGDT. Table 6 demonstrates that WGBF effectively propagates useful information across frames, thereby enhancing the model’s capability to capture spatiotemporal information.
4. The Impact of Sequence Length. We evaluated the influence of sequence length on reconstruction performance using different numbers of frames. Figure 9a shows that PSNR increases with sequence length, but the improvement becomes marginal beyond 12 frames, while the computational cost rises significantly. Therefore, a sequence length of 12 is adopted as a trade-off between reconstruction performance and computational efficiency.
5. Effect of Diffusion Steps. We evaluated the effect of the diffusion step count by testing six variants with T∈[1,2,4,8,16,32], and the results in Figure 9b show that the model produces almost no useful prior features when T=1, which leads to a clear drop in reconstruction quality. As the value of *T* increases, model performance improves; however, the number of iterations reaches 8, the curve basically converges. This indicates that, within the compact latent space, merely eight iterations are sufficient to generate effective prior information, thereby achieving a favorable balance between accuracy and speed.

## 5. Discussion

The results demonstrate that the proposed WGDT-Diff framework enables effective reconstruction of undersampled cardiac cine MRI, particularly under high acceleration factors. Compared with existing methods, WGDT-Diff achieves better reconstruction quality, detail fidelity, and temporal consistency, supporting our hypothesis that cine MRI reconstruction benefits from the joint modeling of frequency-aware decomposition, dynamic spatiotemporal dependencies, and diffusion-based prior learning.

Compared with previous CNN, RNN, and Transformer-based methods, the advantage of WGDT-Diff lies in its explicit separation of low-frequency structural information and high-frequency detail information. The wavelet-guided dynamic Transformer focuses on preserving stable structural representations and temporal continuity, while the diffusion prior compensates for missing high-frequency details. The improved quantitative and qualitative results suggest that such a hybrid design is more effective than relying on regression-based reconstruction alone.

While these results confirm the effectiveness of WGDT-Diff, they also highlight the need to examine reconstruction reliability when diffusion-based priors are introduced, since such priors may potentially generate hallucinated high-frequency details. Based on the qualitative results in Figure 4, Figure 5 and Figure 6, we did not observe obvious non-anatomical structures introduced by the proposed method on the evaluated datasets. At lower acceleration factors, such as 4× and 6×, most methods produce visually acceptable reconstructions. In contrast, under higher acceleration factors, the differences become more pronounced: MoDL shows degradation in reconstruction quality, while KIGAN tends to produce unrealistic shadow-like structures in some regions. By comparison, the proposed WGDT-Diff preserves clearer anatomical boundaries and exhibits more localized error patterns, suggesting that the introduced diffusion prior improves detail compensation without causing obvious structural hallucination in the current setting. Nevertheless, since the present study is mainly based on healthy-subject data, the reliability of the proposed method in pathological cases not represented in the training set remains to be further validated in future work.

Several limitations remain. The current study is mainly based on retrospective undersampling and image-level evaluation, and further validation on larger multi-center datasets and more diverse acquisition settings is needed. Future work will focus on improving inference efficiency, enhancing cross-domain generalization, and evaluating the proposed framework on clinically relevant downstream tasks. Overall, this study suggests that combining dynamic modeling with frequency-aware and generative priors is a promising direction for accelerated cardiac cine MRI reconstruction.

## 6. Conclusions

This paper presents WGDT-Diff, a unified framework for accelerated cardiac cine MRI reconstruction. The proposed method combines a wavelet-guided dynamic Transformer with diffusion priors learning to jointly model low-frequency structural information and high-frequency detail information. Specifically, the diffusion module generates compact latent prior features that are consistent with the distribution of fully sampled data, thereby providing complementary guidance for recovering fine-grained details that are often degraded under undersampling. Meanwhile, the WGDT exploits wavelet-domain decomposition and spatiotemporal modeling to capture stable low-frequency structural representations and temporal dependencies in the image sequence. By integrating these two components, WGDT-Diff improves detail fidelity, structural preservation, and temporal consistency in reconstructed cine MRI sequences.

Experimental evaluations on the CMRxRecon dataset and the in-house dataset verify that WGDT-Diff consistently outperforms existing SOTA methods in terms of reconstruction quality, detail recovery, and temporal coherence, particularly under high acceleration settings. These findings demonstrate the effectiveness and robustness of the proposed framework and suggest its potential value for accelerated cardiac cine MRI reconstruction.

## Figures and Tables

**Table 1:** Dataset Partition from Different Sources.

Set Type	Dataset	Coils	People/Slices/Frames	Count
Train	Cine-Sax	Single	27/6/15	2430
Validation	Cine-Sax	Single	7/6/15	630
Test	Cine-Sax	Single	7/6/15	630
	Cine-Sax*	Single	9/6/12	648
	Cine-Lax*	Single	18/3/12	648

**Table 2:** Quantitative comparison with various models on the in-house (Cine-Sax) dataset. Best results are in **bold**.

	Method	4×			6×			8×			10×		
		PSNR↑	SSIM↑	NMSE↓	PSNR↑	SSIM↑	NMSE↓	PSNR↑	SSIM↑	NMSE↓	PSNR↑	SSIM↑	NMSE↓
Baseline	ZF	33.36	0.8655	0.4285	27.00	0.6693	2.1659	25.31	0.6210	3.3995	24.46	0.5930	4.0082
Static	MoDL	36.26	0.9081	0.1857	31.81	0.8124	0.5198	29.79	0.7701	0.8438	28.89	0.7491	1.0411
	KIGAN	34.39	0.9090	0.2928	31.06	0.8234	0.6386	29.47	0.7670	0.9087	28.25	0.7366	1.2302
	SwinIR	36.31	0.9025	0.1854	31.45	0.8215	0.5666	29.35	0.7868	0.9322	28.51	0.7602	1.1658
	DiffRecon	35.70	0.8961	0.2186	30.94	0.8124	0.6543	29.09	0.7715	0.9863	28.36	0.7428	1.2082
Dynamic	CRNN	35.22	0.8965	0.2418	30.25	0.7984	0.7754	28.76	0.7574	1.0525	27.50	0.7243	1.4308
	VRT	36.41	0.8973	0.1808	31.53	0.8097	0.5575	29.51	0.7813	0.9051	28.82	0.7597	1.0478
	Ours	**37.02**	**0.9338**	**0.1458**	**32.31**	**0.8347**	**0.4773**	**30.71**	**0.8231**	**0.7768**	**30.27**	**0.7909**	**0.8344**

**Table 3:** Quantitative comparison with various models on the CMRxRecon (Cine-Sax*) dataset. Best results are in **bold**.

	Method	4×			6×			8×			10×		
		PSNR↑	SSIM↑	NMSE↓	PSNR↑	SSIM↑	NMSE↓	PSNR↑	SSIM↑	NMSE↓	PSNR↑	SSIM↑	NMSE↓
Baseline	ZF	30.77	0.7961	0.5852	23.65	0.5286	3.1469	21.54	0.4691	5.1498	20.26	0.4269	6.7474
Static	MoDL	34.31	0.8866	0.2474	30.19	0.7627	0.6368	28.12	0.7042	1.0199	27.18	0.6839	1.2672
	KIGAN	31.93	0.8534	0.4443	27.61	0.7065	1.1806	25.33	0.6181	2.0000	23.44	0.5716	3.2068
	SwinIR	34.17	0.8832	0.2571	29.40	0.7819	0.7606	27.71	0.7391	1.1296	26.58	0.7073	1.4517
	DiffRecon	34.42	0.9024	0.2512	30.49	0.8096	0.6082	29.08	0.7853	0.8343	27.88	0.7537	1.1102
Dynamic	CRNN	33.01	0.8724	0.3343	28.46	0.7454	0.9483	26.71	0.6888	1.4139	25.78	0.6656	1.7461
	VRT	33.84	0.8760	0.2826	28.38	0.7387	1.1309	27.76	0.7329	1.1201	26.57	0.7036	1.4811
	Ours	**35.60**	**0.9210**	**0.1766**	**31.76**	**0.8688**	**0.4807**	**30.03**	**0.8024**	**0.8023**	**28.70**	**0.7520**	**0.9659**

**Table 4:** Quantitative comparison with various models on the CMRxRecon (Cine-Lax*) dataset. Best results are in **bold**.

	Method	4×			6×			8×			10×		
		PSNR↑	SSIM↑	NMSE↓	PSNR↑	SSIM↑	NMSE↓	PSNR↑	SSIM↑	NMSE↓	PSNR↑	SSIM↑	NMSE↓
Baseline	ZF	28.84	0.6908	1.8788	22.50	0.4868	6.6528	20.91	0.4596	9.6300	19.26	0.4167	12.3187
Static	MoDL	34.12	0.8031	0.4597	30.47	0.6874	0.9898	28.65	0.6488	1.5048	27.14	0.6269	2.0751
	KIGAN	30.56	0.7341	1.2099	26.76	0.6088	2.6908	24.54	0.5002	4.3056	23.36	0.4630	5.3290
	SwinIR	34.40	0.8867	0.3949	30.24	0.7898	1.0073	27.94	0.7319	1.6901	26.79	0.7029	2.2353
	DiffRecon	35.05	0.9210	0.3424	32.03	0.8582	0.6559	30.39	0.8161	1.3636	29.33	0.7885	1.5992
Dynamic	CRNN	32.98	0.8540	0.5564	29.10	0.7310	1.3129	27.28	0.6886	1.9677	26.27	0.6660	2.5464
	VRT	34.08	0.8812	0.4438	29.35	0.7671	1.4358	28.38	0.7609	1.4720	27.12	0.7261	2.0435
	Ours	**36.59**	**0.9304**	**0.3120**	**32.63**	**0.8634**	**0.5966**	**30.83**	**0.8197**	**1.1137**	**29.63**	**0.8076**	**1.3633**

**Table 5:** Quantitative comparison of SOTA reconstruction methods in terms of the number of parameters and inference time.

Method	MoDL	KIGAN	SwinIR	DiffRecon	CRNN	VRT	Ours
Params (M)	1.14	109.72	64.20	164.31	2.98	54.98	12.04
Inference time (ms)	45	125	83	628	120	179	305

**Table 6:** Quantitative results of ablation studies on various variants with AF = 6× on the Cine-Sax dataset. Best results are in **bold**.

Variant	Components			Metrics		
	Diffusion	Wavelet Transform	WGBF	PSNR↑	SSIM↑	NMSE↓
w/o DM	×	✓	✓	31.22	0.7881	0.7524
w/o WT	✓	×	✓	31.94	0.8147	0.6383
w/o WGBF	✓	✓	×	30.85	0.8017	0.8741
WGDT-Diff	✓	✓	✓	**32.31**	**0.8347**	**0.4773**

## Data Availability

The publicly available dataset CMRxRecon used in this study can be found here: (https://github.com/CmrxRecon/CMRxRecon2024, accessed on 3 May 2024). The anonymized partition index used in the experiment can be viewed at https://github.com/lunisgod/WGDT-Diff, accessed on 26 April 2026.
